# Puerperal Infection1Read before the Buffalo Obstetrical Society, May 24, 1892.

**Published:** 1892-08

**Authors:** Eugene A. Smith

**Affiliations:** Buffalo, N. Y.


### PUERPERAL INFECTION.[1]

By EUGENE A. SMITH, M. D., Buffalo, N. Y.

In a former paper read before this society, entitled Puerperal Fever, the febrile condition not due to septic infection, which complicate the lying-in period, were discussed. In this paper I shall endeavor to excite a discussion of the etiology of puerperal fever due to septicemic processes, and more properly named puerperal septicemia.

Omitting all discussion of predisposing causes, it may be stated that the exciting cause of puerperal septicemia is the lodgement of pathogenic bacteria in the maternal secretions and tissues, and, therefore, we have to discuss their modes of entry.

From the teaching of some men who hold that all cases of puerperal septicemia can be prevented, it follows that the obstetrician is wholly blameworthy who finds one of his patients afflicted with septic invasion. I well remember the fear and trepidation with which I watched my first puerperal cases after studying the extreme views expressed by Dr. Garrigues in his book entitled Antiseptic Midwifery. Since then I have seen that the most careful physicians do meet with cases of puerperal sepsis, and usually these cases have developed just where the obstetrician was most observant of antisepsis, or had least reason to expect such trouble. In hospital as in private practice, antiseptic measures have nearly exterminated childbed fever, yet the spectre still stalks abroad occasionally in the practice of even the most radical advocate of antiseptic care during the puerperium.

If, then, we decide that rigid asepsis and antisepsis do not always insure against the septic infection of lying-in patients, it follows that the physician is not always responsible for the entry of pathogenic bacteria, and the question arises as to how such bacteria gain access to the system.

The external infection of patients, or the introduction of a poison by unclean hands, instruments, or clothing, was pointed out by Semelweis, of Austria, and in the course of the last fifty years antiseptic midwifery has developed from this teaching of a man who was held in contempt during his life for being the pioneer in this field.

In the infection of a patient from external sources, where the physician's hands and instruments are absolved, the blame must fall on nurse or surroundings. What patients may and do escape

1. Read before the Buffalo Obstetrical Society, May 24, 1892.

when an ignorant nurse has charge of the puerperal bed is wonderful. In a case of mine, the attendant nurse, a midwife of the old school, left the washing of the dinner dishes, dried her hands on a dirty towel, dipped her fingers in a lump of lard, and proceeded to examine the patient to find the progress of the case. To further point the moral in this instance, a case of severe sepsis should have followed, but unfortunately for the moral, the patient had no trouble.

Such experiences in the surroundings of puerperal cases make some men careless, until the fact is startlingly brought before them that uncleanliness in the physician is much more liable to cause septic infection than the same fault in the midwife. The doctor is daily in contact with pathogenic bacteria, the streptococci and staphylococci of pus and erysipelas. The uncleanly midwife may infect her patients with non-pathogenic bacteria indefinitely; the bacterium term, for instance, the putrefaction, which causes fetor. But once having a case of sepsis, the midwife is more likely to have several more, because she does not understand the contagion she carries.

Dr. P. W. Van Peyma draws attention to the fact that patients may infect themselves by the involuntary application of their hands to the genitals in the agony of delivery, and he believes this to be the probable etiology of many cases of puerperal sepsis in women who have had no attendant during labor.

When the external introduction of the poison can be excluded, how are cases of puerperal septicemia to be explained? This question brings up the subject of mixed infection, or anto-infection, a subject which has several times been mentioned, but not discussed in our society. In a former paper, I related in full the history of a case of septic peritonitis occurring in the General Hospital, and ending fatally five days after labor. The post-mortem revealed double pyosalpinx, with a normal aseptic condition of uterus and vagina. The patient was a prostitute, with a history of gonorrhea.

From this case and similar ones, we learn that the gonococci, though they are not pyogenic, still do excite inflammatory changes which make a nidus for the development of pathogenic, pyogenic bacteria, entering by some superficial distant abrasion of skin or mucous membrane, and circulating in the blood. Lately I assisted in extirpating purulent buboes from a girl of eighteen, who was two months pregnant, and had gonorrhea and possibly chancroids. The lymphatics leading from the vagina and vulva are capable of carrying the poison to the intrapelvic as well as to the extrapelvic chains of lymphatic glands. Imagine this case to be nine months advanced and the mechanical violence of labor to disturb some intrapelvic pregnant suppuration, and we readily see the cause for a case of puerperal septicemia.

In hospital obstetric practice I have learned to watch carefully all cases of leucorrhea. Owing to the presence in the diseased mucous membrane of pathogenic bacteria, notably the gonococci and pyogenic microörganisms, patients are prone to post partum septic troubles, as their babies are to ophthalmia neonatorum.

In this way also cases of suppurative inflammations, intestinal ulceration, ulcerative endocarditis, diphtheria, and erysipelas, complicating the puerperal state, may be shown to influence the onset of septic processes in the maternal passages, by the spread of pyogenic bacteria from the spot of suppuration to the injured susceptible spots in the genital tract after delivery.

In a case of rheumatic endocarditis, complicating the lying-in period, reported in a former paper, I drew attention to the happy effects following the internal use of salicylate of sodium, and the local use of douches and chloride of zinc for diphtheroid patches in the vagina. Here was a case of infection of the inflamed endocardium by streptococci and staphylococci entering the blood, and a secondary infection of the wounds in the vagina. Conversely in a second case, giving a rheumatic history, I found phlebitis in the groin and thigh, due to some septic inoculation, possibly gonorrheal, followed by endocarditis. Here the temperature ranged above 104° F. for four days, but as the attack did not develop until the second week after delivery, she was able to throw off the poison and recovered.

I have not seen a case of erysipelas in the puerpera cause puerperal septicemia, but in the following case I saw the converse :

Mrs. B., aged 30, primipara, has a husband who was treated by me just before his marriage for gonorrheal and chancroidal suppurating buboes. I found his wife in labor upon my first visit, and she gave a history of an attack of facial erysipelas two years before marriage, and a troublesome leucorrhea beginning shortly after marriage. Her baby was delivered in the evening with forceps, and a laceration of the perineum occurred. On the next morning she had fever, and it continued for five days, when a violent attack of facial erysipelas reinforced the puerperal sepsis already existing as an endometritis and endocolpitis. The peritoneum and parametrium were not involved, and she made a good recovery.

Whether she had an erysipelas of the genital tract, or the septic infection merely lighted up dormant streptococci erysipelatosm, would be a question for a bacteriologist to debate, but here was a case of mixed infection which primarily was the sin of the husband who contracted gonorrhea by illicit intercourse, and secondly, was made possible by an attack of erysipelas in the wife before marriage. I may say that I was not treating outside cases of erysipelas, and my contemporary obstetric cases progressed favorably.

In regard to phthisis, pneumonia, typhoid fever, syphilis, scarlatina, and other diseases, caused by pathogenic but not pyogenic bacteria, it may be said that they make the puerpera more liable to puerperal septicemia by debilitating the system so that pyogenic germs can more readily flourish upon entering the secretions and wounded tissues externally, or reaching the vulnerable spots by the circulation internally.

In conclusion it might be of interest to discuss the terms anto-infection and previous or mixed infection. Anto-infection signifies that the patient herself originates microörganisms to poison herself, and, therefore, is an incorrect term and should not be used. When used it is intended to show that the physician considers his patient was not infected by hands, instruments, or surroundings, but developed the puerperal septicemia from a source of infection within the patient which he could not control.

It would be better, however, to call such conditions the result of a previous or mixed infection, and then strive to discover the point of entry of pyogenic microörganisms in order that a rational and radical treatment of the source of infection could be carried out, if possible.

With more care in the examinations of patients with puerperal sepsis, and a more thorough inquiry into the etiology, the physician can be more scientific in treatment, and can prove himself blameless when he has used all aseptic and antiseptic precautions during the stages of labor.

---

Speaking of the life of phthisical patients sent to Colorado, Dr. J. M. Keating, of Colorado Springs, in a paper read before the El Paso County Medical Society, believes that many cases of phthisis which have a fatal ending would have recovered if given rest. He thinks it is a mistake for patients to rush madly into out-door life. He believes that a rest cure, combined with inhalation of oxygenated air, will be one of the best means to combat phthisis. He thinks at the health resorts the cottage system should be used.—*Lanphear's Kansas City Medical Index.*

